# On Diarrhœa, as Seen in the Camps

**Published:** 1865-06

**Authors:** Erwin L. Jones

**Affiliations:** Ass’t. Surg. 53d Wis. Inft.


### CHICAGO MEDICAL JOURNAL.

VOL XXII.] JUNE, 1865. [No. 6.

### ORIGINAL COMMUNICATIONS.

### ON DIARRHŒA AS SEEN IN THE CAMPS.

By ERWIN L. JONES, Ass't Surg. 53d Wis. Inft.

*Messrs. Editors Chicago Medical Journal:—*

I have the pleasure to lay before you for publication, a few words upon the subject of Diarrhœa, and which, if they are worthy, I am confident you will find a place for in your *Journal*, especially as the ideas advanced are based upon those principles of Medicine taught at the College in which you occupy the position of instructors, and of which, I am proud to say, I am a graduate.

I do not propose to advance an elaborate article upon the subject upon which so much has been said—of the cause, treatment, and morbid anatomy of this disease—indeed, that I am not competent to do so, I can say with no discredit, but

I do propose to tell what I have seen myself; to recommend those plans of treatment that have proved the most successful with me; to call to mind old facts and to claim no credit for the discovery of new ones.

Since March, 1864, I have been continually employed as Contract Surgeon by our Government, and doing duty in general hospitals, the greater portion of the time in Nashville, Tenn., and among the great variety of diseases thus brought under my immediate notice, none appeared so frequently, or whose treatment was so unsatisfactory—not only at my own hands, but at those of my medical associates—as the disease under consideration—filling the hospitals with men that, but for this affection, would be able to do duty in the field.

I do not wish to be understood as speaking of this disease as an insignificant or simple one; on the contrary, I wish to urge its great, and I am inclined to think unacknowledged, importance. Often have I heard the medical attendant say to his visitors, "All these patients have *only* a diarrhœa."

"What treatment are they under?" you ask. The answer is, "Treatment does them no good," or "They complain of greater discomfort when their discharges are controlled." And upon inquiry you learn that the way these unfortunate fellows get their "discharges controlled" is by some astringent preparation, placed in the ward for general distribution, with directions to a nurse (who enlisted to destroy life with combustible powder, but being elevated to the position of apothecary, becomes the means of accomplishing the same object with powders, not always combustible but often equally destructive,) to give all patients with diarrhœa a certain amount at certain intervals, of a certain astringent preparation, common to all; treating, or rather maltreating, all upon the assumption that their ailments are similar, and therefore universal benefit must be derived from the same prescription; or in other cases you may find Hope's Mixture, or Camp. et Opii Pil., or some other of the astringents usually employed, prescribed, but all given for a common purpose, i. e., "to *control the discharge*."

Again, may be found some one who does not follow the same routine practice, who has found that in some cases a mild cathartic may be of service in unloading the beseiged bowel of any irritating substance that may have been industriously crammed into it under the name of sanitary purposes, and before the "discharge controlling" practice came into effect; or who accomplish the same by depleting from the portal circulation, etc., and having done this, thinks he has done everything, (and Heaven knows this is better, and not so frequently the case as the other extreme,) and is surprised that the patient does not recover under his remedy (which he continues to ply,) and several well men's rations of half-cooked beans, beef, and sutler's sole-leather pies—of the partaking of which, however, he never knows—never having heard that a man's stomach may not be made a general purveying depot for any substance, and having no rational idea of its capacity.

Many practitioners are not aware that their patients are employing an improper diet, but they *should know and prevent it*, and prescribe the diet (in my opinion the most important part of the treatment,) with as much regularity and care as he does his pills and powders.

Another class condemn all therapeutical measures and contend that dietetic treatment is *all* that is necessary; but from ignorance or inattention, an injudicious selection of the article of diet, is equally unsuccessful with the others.

I do not mean to say that all of these practitioners are unskillful, I would rather think that they are indolent, and the fatigue and difficulty of inquiring into each individual case, when an entire ward full is depending upon his hands for treatment, has a way of reminding one of a comfortable place to sit down, where diarrhœa can not come, that is very hard to resist. Besides, more patients are usually entrusted to the care of surgeons in hospitals, than they can do justice to, as many as one hundred and fifty not unfrequently depending for treatment upon one person. The result is, that the Medical attendant fails to do justice to the patients, himself, or the profession he should strive to improve and cultivate.

Many diarrhœas are due to causes that can not be always removed; of these I shall not speak. I would refer to those disorders occurring from errors in diet, and constituting, in my opinion, a greater proportion than have been generally acknowledged; those too, who have diarrhœa arising from other causes, are often kept in a state of prostration by improper food and medication, long after the first cause has been removed.

I have heard surgeons say, " My patients can not complain of their diet at my hospital; they have all they want." And here is where the error often lies; the patient being the only judge of what he shall eat. But under some circumstances they are not so fortunate. Food of an inferior and unsuitable kind, or if good, spoiled by the inefficiency of cooks who, so far as my experience extends, are enlisted men, with no knowledge of that branch of industry, and who do not remain in the situation long enough to profit by what they learn there, but are sent to the field and their places in turn supplied by others, who are themselves deposed by some examining board or by an order to send all men able for duty to their respective commands.

I think it would be a measure of economy if our Government would procure efficient cooks, even if obliged to employ them as such, and have at least one in every general hospital.

I am inclined to think that many diarrhœas are kept up by a state of irritation which, if allowed to go down, the patient would be restored. I can say that I have cured many patients by simply regulating the diet, carefully noting such substances, in each individual case, as appeared to agree the best with them, and excluding all others. Employing as few articles at a time as possible, at the same time attending to the general condition of the system, and surrounding the patient with those hygienic influences, so truly important alike to the healthful and infected.

I found some done well on nitorginous food, others on farinaceous or oleaginous, but the latter less frequently produced good results. Some were totally unable to take food at all, the stomach rejecting at once any and everything taken into it, or only after a time of nausea and oppression, accompanied by griping pains in the bowels; in such cases I enforced total abstinence, allowing the patient to take nothing into his stomach until all such symptoms had entirely passed away, and was then gratified to find that some article of food would be acceptable if given in proper quantities and properly prepared, while the pain, nausea, and oppression so frequently complained of before, were lessened or totally destroyed.

For four months last summer I was ill with the disease myself, during which time, not only myself but all my medical associates industriously contributed to my misery, and the amount of medicine I swallowed is truly fabulous. Everybody had a formula that was a sure one, and congratulated me on my good fortune in falling into their hands for treatment, while I regretted that the same had not sooner taken place.

At last I discontinued all medication, and the sense of discomfort in my abdomen taught me what I could *not* eat, and suggested the propriety of thinking of something that I *could*, and from that date I began to recover, and did so entirely in a reasonable time.

At times I found that I could not eat the least particle of farinaceous food without severe griping pain, while animal soups and even meats properly prepared, would occasion no discomfort. I could even eat baked beans, while wheat bread *always* caused pain. In other cases that afterwards occurred in my practice there, I noticed the same peculiarity.

I suppose this was occasioned by the difference in the seat of the irritation, which exists where certain elements of food were digested, therefore such articles as contained these elements always produced pain.

When I received a patient for treatment with Diarrhœa, I removed all substances from the intestinal canal by a mild but thorough cathartic; having done this, I found what articles of food would be best received and in what quantities. If much irritation of the stomach existed, the usual means were employed to relieve it, small pieces of ice swallowed always proving grateful to the patient. In the greater number of cases stimulant treatment was necessary, as soon as the irritability was sufficiently allayed by rest, both mechanical and physiological, and usually whiskey, or in other cases mur. tinc. ferri was the prescription, stronger astringents than these I never found necessary. Sometimes opium to allay pain in the bowels, but I generally found that if a proper diet was chosen, no pain was produced.

When the disease was characterized by remissions quinine was used, frequently in combination with piper nigra pulvis.

The most scrupulous attention to cleanliness of person, clothing, and everything surrounding the patient, fresh air, and a uniform temperature, meals at regular hours, and in proper quantities, and consisting mostly of soups, solid food being excluded as much as possible.

At home I have a perfect history of my own and many other cases, that I could produce if necessary, to show the value of this treatment, but as I write from the field I can not do so at present.

